# Promoting Adolescent Healthy Relationships (The About Us Program): Protocol for a Randomized Clinical Trial

**DOI:** 10.2196/30499

**Published:** 2021-09-01

**Authors:** Pamela Anderson, Karin Coyle, Stephanie Guinosso, John L Ferrand, Arthur Owora, Rebecca F Houghton, Eric Walsh-Buhi

**Affiliations:** 1 ETR Associates Scotts Valley, CA United States; 2 Department of Applied Health Science School of Public Health Indiana University Bloomington, IN United States; 3 Department of Epidemiology and Biostatistics School of Public Health Indiana University Bloomington, IN United States; 4 School of Public Health San Diego State University San Diego, CA United States

**Keywords:** adolescents, youth, teens, healthy relationships, unintended pregnancy, teen pregnancy, sexually transmitted infections, sexually transmitted diseases, sexual health education, school-based health center, randomized controlled trial

## Abstract

**Background:**

Romantic relationships play a critical role in adolescent development, and by middle adolescence, most young people have been involved in at least one romantic relationship, a context in which most sexual interactions occur. Research suggests adolescents lack positive models and skills related to building healthy relationships.

**Objective:**

This project aims to test the impact of an innovative healthy relationships intervention, called About Us, implemented in school-based health centers (SBHCs) in California in a randomized controlled trial.

**Methods:**

About Us is being tested using a 7-site, 2-group, parallel randomized controlled trial with a treatment versus control allocation ratio of 3:2 to assess the impact of the intervention relative to the standard of care among adolescents aged 14 to 18 years. Adolescents with active parental consent provide study assent at each of the 3 survey time points: baseline, 3 months postintervention, and 9 months postintervention. A stratified randomization procedure was used to ensure balance in key covariates and screening criteria across intervention groups. Through benchmark intent-to-treat analyses, we will examine the primary outcome of this study—the impact of About Us relative to the standard of care 9 months following the end of the intervention on the prevalence of vaginal or anal sex without condoms in the past 3 months. The secondary outcomes are four-fold: what is the impact of About Us relative to the standard of care 3 and 9 months following the end of the intervention, on (1) the prevalence of abstinence from vaginal or anal sex in the past 3 months, (2) composite scores of relationship communication and positive conflict resolution among participants involved in a relationship at baseline, (3) the prevalence of SBHC service use or information receipt in the past 3 months, and (4) composite scores of condom use intentions and attitudes regarding condoms and other birth control? Additionally, as part of our sensitivity analyses, two additional analyses will be implemented: modified intent-to-treat and complete case analysis.

**Results:**

This project (ClinicalTrials.gov #NCT03736876) was funded in 2016 through the Family Youth Services Bureau as part of the Personal Responsibility Education Innovative Strategies program. Baseline data collection took place between February 2018 and March 2020, yielding a total of 5 cohorts and 533 study participants: 316 assigned to treatment and 217 assigned to control. Ongoing follow-up data collection continued through May 2021.

**Conclusions:**

About Us draws on developmental science to create a contextually and developmentally relevant program that addresses motivation and emotional influences in sexual decision-making. The intervention was designed for implementation within SBHCs, an understudied venue for relationship and sexual health promotion interventions. Unfortunately, COVID-19 pandemic restrictions led to school closures, interrupting ongoing programming, and in-person follow-up data collection, which has affected study attrition.

**Trial Registration:**

ClinicalTrials.gov NCT03736876; https://clinicaltrials.gov/ct2/show/NCT03736876

**International Registered Report Identifier (IRRID):**

DERR1-10.2196/30499

## Introduction

### Background and Rationale

Romantic relationships play a critical role in adolescent development [1]. By middle adolescence, most young people have been involved in at least one romantic relationship [2], a context associated with greater odds of sexual intercourse [3] and in which most sexual interactions occur [4]. Research suggests adolescents lack positive models as well as norms and skills related to building healthy relationships [5]. Nonetheless, developmental psychologists emphasize that adolescence represents a new period in which past models of relationships may be reshaped, priming young people for healthier adult relationships [6]. Additionally, research suggests adolescents’ views on relationships may be influenced by discussions with peers [7], highlighting the importance of promoting positive normative beliefs and sexual behavior within the context of relationships.

Opportunities for sexual experimentation and status attainment are often at the forefront of adolescents’ initial views of romantic relationships [8], providing leverage points for interventions to guide adolescents in setting sexual boundaries and identifying potentially unsafe sexual situations. Accordingly, adolescents with experience in relationships are primed for prevention programs that address critical relationship skills, such as communication in intimate relationships and navigating different sexual boundaries. Using a targeted approach that involves identifying and engaging adolescents with increased vulnerabilities maximizes resources and prioritizes serving them. This project centers on promoting healthy relationships and expanding the typical prevention education foci in sexual health.

Focusing on romantic relationships aligns with developmental science underscoring the importance of relationships in adolescence. Because of continued disparities in sexually transmitted infections (STI) and unintended pregnancies in the United States, there remains a need for addressing pregnancy and disease prevention. Among adolescents aged 15 to 19 years, the teen birth rate declined 7% from 2017 to 2018, from 18.8 to 17.4 births per 1000; however, the teen birth rate remained approximately twice as high for Hispanic and Black teens compared to non-Hispanic White teens [9]. Additionally, 75% of teen pregnancies are unintended [10,11]. Further, rural counties observe higher teen birth rates compared to urban and suburban areas [12]. Very few evidence-based program models addressing sexual risk have been developed and tested with targeted populations, such as Hispanic adolescents or those residing in more rural areas [13,14].

Not all young people are at equal risk of experiencing an unplanned pregnancy or STI. Indeed, some contextual factors, such as experience with romantic relationships or even exposure to violence, place adolescents at greater risk for engaging in sexual activity, including unprotected (eg, condomless) sex that could lead to an unplanned pregnancy or STI. In addition, exposure to violence, both directly and indirectly, is associated with risky behaviors (eg, unprotected sex), lack of self-efficacy, anxiety, depression, challenges developing and maintaining healthy relationships with prosocial peers, and increased associations with peers who endorse unsafe norms and behaviors [5,15]. Estimates suggest that 60% of children and adolescents younger than 18 years have been exposed to at least one form of violence in the previous year [16], meaning these adolescents are at increased vulnerability for other poor outcomes.

Most existing evidence-based programs (EBPs) for adolescents are delivered in community-based settings or schools (eg, Making Proud Choices; Reducing the Risk) [17,18]. A few are delivered through health clinics (eg, Seventeen Days) [19], but none to our knowledge have been developed and tested expressly for implementation via school-based health centers (SBHCs). SBHCs are clinics initially created in response to adolescent health needs that operate on or near school campuses and provide a range of age-appropriate health-related services to adolescents. Today, most SBHCs also offer a comprehensive array of services, including primary care, mental health services, and health education. In most high school SBHCs, reproductive health is a core service [20]. As SBHCs become more common across the United States, the role SBHCs play in prevention and health promotion interventions will also grow in importance [21]. SBHCs are uniquely positioned, both physically and philosophically, to reach young people. SBHCs’ location on or near a school campus offers easy access to large groups of adolescents. More importantly, however, SBHCs focus on building trust and meeting young people “where they are” developmentally. Thus, SBHCs offer a unique opportunity to deliver health interventions that integrate prevention education and clinical care [20].

This original paper outlines the rigorous evaluation of About Us, an innovative healthy relationships intervention implemented in SBHCs to reduce the prevalence of unprotected sex and promote stronger relationships among adolescents facing disparities in sexual health outcomes.

### Intervention

Developed from a piloted intervention, About Us is an innovative healthy relationships intervention that promotes positive adolescent romantic relationships, condom use, and highly effective contraceptives if participants are having sex. The program includes 10 lessons (2 lessons are 30-45 minutes long and 8 are 50 minutes long) that blend group-based activities with online activities implemented in a small group format with students in grades 9 or 10 who have parental consent and provide assent to participate.

About Us draws on the latest research on developmental neuroscience to shape content and strategies. Part of the innovative design of About Us stems from the use of positive youth development (PYD) principles and adolescent development literature as core elements that are foundational to the curriculum. PYD is a strength-based approach used to promote adolescents’ prosocial competencies and skill-building related to their positive health and well-being [22]. The adolescent development literature, such as that reported by Collins [23], guides the relationship development content and helps ensure it is age-appropriate.

The program also draws on dual-process theories to address socioemotional well-being and cognitive influences on sexual decision-making [24]. For example, in a lesson on correct and consistent condom use, the program addresses adolescents’ explicit intentions to use a condom during sexual intercourse and has them explore which circumstances might precipitate their decision to have sexual intercourse without condoms, prompting them to recognize and navigate these experiences to avoid condomless sex. Finally, the program draws from social cognitive theory [25], both in terms of key constructs in skill acquisition, such as building self-efficacy or confidence in one’s ability to perform a behavior and shaping the process of learning through observational learning or modeling during instruction. Bandura [25] posited that self-efficacy is influenced in 4 ways: mastery experiences (successful completion of a task), modeling (observing others similar to oneself perform a task successfully), social persuasion (information from others that one can perform a task successfully), and physiological arousal states (information from one’s physiological state, such as anxiety). The program draws on these strategies in the skills-based sessions to maximize its impact on self-efficacy. For example, to apply the concept of mastery experiences, the program includes role-play exercises in which adolescents practice in context (small groups and online) and receive feedback on their use of the skills.

Each About Us session includes an initial soap opera-like story to build interest and illustrate key concepts, 2-3 group-based activities with reflection, individual app-based work on computer tablets (to allow for personalization of the content), and a group-based debrief to reflect on the session and reinforce key messages. To maximize the relevancy of the content and strategies, we engaged adolescents in developing the About Us curriculum, and they contributed to its naming, cast of characters, and storylines.

For this study, the group-based content was delivered by trained facilitators (eg, health educators) from participating SBHCs. The app-based content was housed on a secure website and accessed through tablets with unique logins for each participant. Online activities were completed individually during each session (eg, completing a poll or watching and responding to a brief video). Several lessons also included homework activities that encouraged communication between students and a caring adult. Additional details of About Us are displayed in Table 1.

Participants assigned to the treatment group attended group sessions at the designated space. Health educators followed up with students who missed a session to engage them and remind them of the next session. Participants assigned to the treatment group received a US $30 incentive if they attended 6 or more sessions.

**Table 1:** About Us intervention components.

Component	Amount, duration, and intended dosage	Content	Who delivers	Setting
Group-based sessions	10 (2 prelessons and 8 regular lessons) over 4-9 weeks for a total of 10 hours of programming.	Characteristics of healthy and unhealthy relationships, communication skills (having difficult conversations, such as sexual consent, sexual boundaries, and condoms; using “I” statements); personal and sexual boundaries and sexual consent; condom and contraceptive use; influences on sexual decisions in relationships; ending relationships.	Trained health educators from the school-based health centers.	During school (students pulled out of class to come to the health center or other agreed-upon space on the school campus).
Online work during regular group-based sessions	Approximately 15 minutes in each regular lesson; students are able to revisit content from prior lessons outside of group sessions during the implementation period.	Same as above; online activities allow for the review and application of key concepts and the personalization of content for each lesson.	Trained health educators from the school-based health centers will prompt and support students using the tablets and the application during the group sessions.	Same as above.
Parent/other adult-adolescent homework	2 homework activities.	Brief conversation-based homework activities focused on healthy relationship values and influences related to sexual expectations in relationships.	Trained health educators “assign” homework activities as part of the group-based sessions; adolescents are asked to bring back a sign-off sheet acknowledging they completed the activity, which will be included in the implementation log.	These were assigned during the program implementation, but the setting for completion was out of school.

### Study Objectives

The overall goal of this project is to test the impact of the About Us blended learning healthy relationships intervention, implemented in SBHCs in a randomized controlled trial (RCT), on reducing unintended pregnancies and STI in adolescents facing disparities in sexual health outcomes.

### Research Questions and Hypotheses

1. During the 9 months following the end of the program, what is the impact of About Us relative to the standard of care on the prevalence of vaginal or anal sex without condoms in the past 3 months? We hypothesize that at the final follow-up, the prevalence of self-reported unprotected vaginal or anal sexual intercourse (ie, without condoms) in the past 3 months will be lower among adolescents in the intervention group than students in the control group.
2. During the 3 months and 9 months following the end of the program, what is the impact of About Us relative to the standard of care on (1) the prevalence of abstinence from vaginal or anal sex in the past 3 months, (2) composite scores of relationship communication and positive conflict resolution among participants involved in a relationship at baseline, (3) the prevalence of school-based health center service use or information receipt in the past 3 months, and (4) composite scores of condom use intentions and attitudes regarding condoms and other birth control? At each follow-up, we hypothesize that, compared to the control group, students in the intervention condition will have: (1) a higher prevalence of sexual abstinence in the past 3 months, (2) higher composite scores showing stronger relationship communication and more positive conflict resolution (3) a higher prevalence of SBHC services utilization or information receipt, and (4) higher composite scores showing stronger intentions and more positive attitudes regarding condom use and other forms of birth control.

## Methods

### Study Design Overview

**Figure 1:**
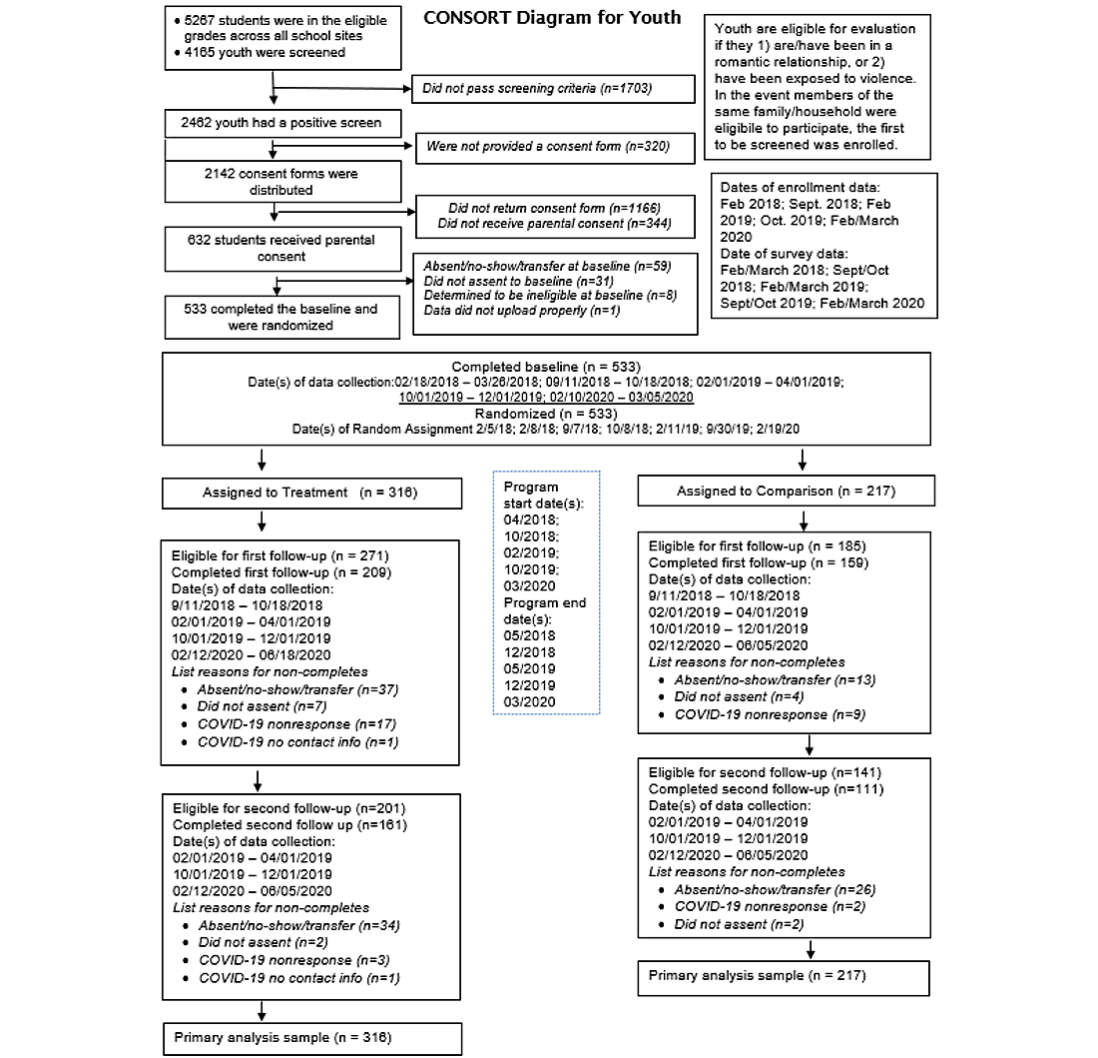
CONSORT diagram for the About Us evaluation (as of October 2020).

This study is a 7-site, 2-group, parallel RCT with a treatment versus control allocation ratio of 3:2, assessing the impact of the About Us program relative to the standard of care among adolescents aged 14 to 18 years. Figure 1 summarizes study screening, eligibility assessments, enrollment, randomization, and follow-up results.

### Study Funding and Ethics Approval

This study was funded in 2016 through the Family Youth Services Bureau (FYSB) as part of the Personal Responsibility Education Innovative Strategies (PREIS) program as an award to ETR. This project was approved by the San Diego State University (SDSU) institutional review board (IRB) in March 2017. In June 2020, Indiana University approved an IRB reliance (protocol 2004100675) on the SDSU IRB approval (protocol HS-2017-0121). The study is registered with clinicaltrials.gov (NCT03736876).

### Study Setting

This project was implemented in 7 SBHCs in rural or suburban communities throughout California with large Hispanic populations. The California School-Based Health Alliance (CSHA) assisted the About Us project team in recruiting participating sites and supporting the clinic screening process used as part of assessing eligibility. The CSHA supports the entire network of SBHCs in California.

### Eligibility Criteria

Students in the 9th and 10th grades between the ages of 14 and 18 years were eligible to participate if they met at least one of two eligibility criteria: (1) had ever witnessed a serious injury or homicide or (2) had a girlfriend, boyfriend, or partner before or during the study consent process (prior or current experience with a romantic relationship). In addition, a student would be considered ineligible if a sibling or step-sibling had already enrolled in the study to avoid contaminating participants in different conditions.

### Recruitment

#### Power Analyses

For a statistical power of 80%, to detect a 9% difference (effect size: prevalence ratio=0.74, 95% CI 0.60-0.91 equivalent to an odds ratio [OR] of 0.65 or Cohen’s *d*=0.24) in the prevalence of condomless sex at 9 months between intervention and control group participants with an allocation ratio of 3:2, we needed to have a sample size of 508 and 339 subjects in the intervention and control groups, respectively, based on a simple two-tailed proportion difference test at a Type I error of 5%.

We selected a minimum detectable effect of Cohen’s *d*=0.2. The rationale for this selection is two-fold: (1) in our R21 pilot test of a less robust version of the About Us curriculum with a very small (<100) sample, we achieved an estimated effect size of *d*=0.15 for our unprotected sex outcome, and (2) in a meta-analysis of group-based pregnancy or STI interventions, the average effect size across multiple studies was OR 0.70, which corresponds to a small Cohen’s *d*=0.19 [26,27].

#### Screening

Study sites administered a grade-wide or school-wide screening questionnaire to assess adolescents’ exposure to violence and relationship status (two of the eligibility criteria noted above). For screening, sites used a variety of customized approaches based on their school protocols. All of the sites opted to include additional items on their screening questionnaires to learn more about the general health and well-being of 9^th^ or 10^th^ grade students (eg, “Do you have a primary doctor?” or “Do you eat fruits and vegetables daily?”).

#### Consent and Assent Process

Per California law, we sought active parental consent for study participation through either a signed copy of the consent form or verbal confirmation via telephone calls (by research staff) when there was a parent signature, but the permission checkmark was ambiguous. Consent forms were available in both English and Spanish. The consent process began in one of two ways: (1) either an in-class presentation about the study was delivered by an SBHC representative or (2) students who met the screening criteria were sent invitations through a required class at school to attend an informational event, where, if interested, they received more information about the study, and parental consent forms were distributed. A single high school differed from other sites in that it provided consent forms to students ahead of the screening procedure. Students were informed that they would receive a US $10 incentive for returning a parental consent form regardless of their parent's decision. They were directed to return parental consent forms to their respective SBHC.

For students whose parents provided study consent and met the screening criteria, site liaisons compiled an eligibility log with the following data: student’s last name, first name, and middle initial; student ID (optional); student’s sex assigned at birth; student’s reported gender identity; the number of screening criteria met (selected from a dropdown menu); school (selected from a dropdown menu); agency (selected from a dropdown menu); whether students have a sibling or step-sibling at the school; and whether that sibling participated in About Us in the past (criterion for exclusion). Evaluation data collectors (EDCs) used this log onsite to determine which students were fully eligible for the assent process.

The study’s EDCs administered the study assent process on the day of the baseline survey administration at a given site. At the time of assent, prospective participants of consenting parents who met all the other eligibility criteria and agreed to study participation were enrolled in the study.

### Baseline Data Collection

At the time of assent and prior to randomization, adolescents completed the baseline survey on a self-administered tablet device via the Qualtrics Offline Surveys application (version 1.4.06) [28]. Following survey administration, EDCs promptly uploaded responses to the QualtricsXM server as soon as a secure internet connection could be achieved. EDCs were provided Verizon Jetpack MiFi devices (7730L and 8800L) for this purpose.

Participants were enrolled and completed baseline surveys on a rolling basis during the spring and fall semesters of 2018 and 2019 and spring of 2020. The targeted enrollment was approximately 173 students per site over 5 semesters (34 to 35 students per semester) for a total of 865 students.

At the completion of baseline surveys for a given cohort at a given site, the EDCs submitted a baseline survey administration log to the evaluation project manager for review and transferred data to the evaluation statistician for randomization into either About Us (intervention) or standard of care (control).

Baseline data collection consisted of two separate instruments: a computer tablet-based survey designed to capture adolescent knowledge, attitudes, and experiences regarding sexual behavior, contraception, STI, and HIV/AIDS; and a brief survey that collected contact information for study follow-up purposes. EDCs were selected and trained by the evaluation team to represent the study in the local communities and schools within which About Us was being implemented and evaluated.

### Randomization

Following baseline survey completion, eligible adolescents who had already received parental consent and assented to study participation were randomized to either the intervention or control group.

To assign participants to study groups (intervention or control), we used a stratified permuted block randomization procedure to ensure balance in key covariates and experience factors across intervention conditions given our need to randomize in smaller cohorts within schools by semester (ie, blocks). Specifically, eligible and consented adolescents were subdivided into strata defined by the sex assigned at birth (male or female) and whether they had one or two of the screening experience factors, followed by permuted block randomization for each stratum. Within each stratum, participants were assigned to either the intervention or control group using a 60/40 split. The goal was to create a balance of sex assigned at birth and experience factors to ensure that the intervention and control groups had an equal distribution of these factors that may affect our primary outcome of interest (ie, having vaginal or anal sex without condoms). In other words, the stratification was done to avoid the potential imbalances or confounding due to the sex assigned at birth and the number of baseline experience factors.

Students randomized to the control group received business as usual (BAU) care only. Students assigned to the control group received no special services beyond BAU provided through the schools. To measure the control group experience, we included a set of general exposure items on the impact survey to assess self-reported dosage from or exposure to teen pregnancy programming or sex education during the study period in the school and community (eg, have you had a guest speaker come to your school to provide any of the following information: abstinence information, sexuality information, pregnancy prevention information, STI or HIV information, etc). We also collected data from our schools (on a “needs and resources assessment”) to evaluate content from BAU education during the study period using a brief, web-based survey collected from our site liaisons and the schools’ health education teachers.

Random assignment duties were limited to the study statistician and conducted within one week of baseline data collection at a particular study site. A participant’s random assignment to the intervention group was communicated to the evaluation project manager, who then transmitted this information to the respective SBHCs study site coordinators.

### Follow-up Data Collection

Follow-up surveys were administered at 2 separate time points following the completion of the About Us intervention (approximately 3 months and 9 months postprogram implementation). Similar to baseline data collection for cohort 1 (spring 2018), cohort 2 (fall 2018), and cohort 3 (spring 2019), most follow-up surveys were conducted in-person on school or SBHC grounds. Upon receipt of a signed assent form, adolescents completed the survey on a self-administered tablet device via the Qualtrics Offline Surveys application and in the presence of an EDC. For any adolescents who transferred or were absent, a follow-up contact protocol was used to locate and provide an opportunity to complete the survey. Specifically, using contact data provided by the adolescent at the baseline or 3-month survey, EDCs would undertake a series of contacts through email, text message, cell phone calls, home phone calls, and/or social media messaging, sending the adolescent a unique link to the follow-up survey. Adolescents received a US $10 incentive for each survey completed up to the end of March 2020.

Follow-up surveys for cohorts 4 and 5 were affected by the COVID-19 pandemic due to school closures and travel restrictions preventing evaluation staff from traveling to study sites to administer the surveys. As a result, the study team adopted an online follow-up protocol as the primary approach to administering follow-up surveys and increased the online survey incentive to US $25, as the literature shows this amount is more effective in increasing response rates [29].

Throughout the study and depending on whether the cohort was surveyed during the pandemic, it was possible for participants in both the intervention and control groups to receive incentives totaling as little as US $10 and as much as US $60. Survey incentives offered to all participants were different than program incentives offered only to those in the intervention group who completed About Us program sessions.

### Measures

Primary measures to be analyzed are listed below in Textbox 1. All secondary measures to be analyzed along with sources are provided in Multimedia Appendix 1 [30-36].

**Behavioral outcomes used for the primary research question.**

**Outcome name:** Condomless vaginal or anal sex in the past 3 months (core measures for Personal Responsibility Education Innovative Strategies grantees) [36].

**Source item(s):**

- When you had vaginal sex in the past 3 months, how often did you or a partner use a condom? Vaginal sex is when a penis is put in a vagina.
- When you had anal sex in the past 3 months, how often did you or a partner use a condom? Anal sex is when a penis is put in a rectum, ass, or butt.
- Original response options for vaginal or anal sex include 1 (all of the time), 2 (some of the time), or 3 (none of the time).

**Constructed measure:**

- Construct a single, dichotomous outcome coded as 1 if the respondent indicated they had vaginal or anal sex without a condom in the past 3 months, 0 if they did not, or missing otherwise.
- To be recoded into a single dichotomous variable (vaginal or anal sex) with response options as follows: 0=all of the time (1), as well as participants who reported not having vaginal or anal sex in the past 3 months; 1=some (2) or none of the time (3); and (?)=missing response for vaginal or anal sex without a condom in the past 3 months.

**Timing of measure:** 9 months following the completion of the program.

### Data Cleaning

#### Phase I

Data cleaning is being implemented in three phases. At the baseline, 3-month, and 9-month surveys, the evaluation team engages in a screening process beginning during data entry and using built-in checks for participant entries via Qualtrics. We are using predefined expectations about normal ranges (eg, aged 14 to 18 years), flagging of dubious data entry and patterns (eg, using prompts to confirm an entry), and skip patterns (eg, if students reported having been in a relationship, they received an additional branch of questions not given to those who never were in a relationship).

After retrieving Qualtrics data from each participant survey from the EDCs, project staff transfers the files to a password-protected shared drive. Participant data are transferred and warehoused in SPSS (version 27; IBM) by data wave (ie, baseline, 3 months, and 9 months). Using SPSS, we are recoding variables, creating new variables (eg, check-all variables, such as race and sexuality, recoded into one categorical variable), and labeling and formatting variables for analysis.

For each data wave and merged data sets, data are checked to ensure they meet predefined range expectations, logical skip patterns, and consistency checks and missing data patterns by examining variable descriptive summary statistics (eg, minimum, maximum, mean, median, and SD), frequency distributions, cross-tabulations, and graphical explorations of variable distributions (eg, box plots, histograms, and scatter plots). Data are also checked against expected data collections (based on the number of randomized participants, lags between baseline, and follow-up surveys) and errors in transferring data from Qualtrics (eg, duplicate entries and inadvertent deletions). This process is implemented using SPSS and SAS (version 9.4; SAS Institute).

#### Phase II

At this phase, we are going back to the original Qualtrics data files for any inconsistent data points and patterns to verify entries and add justifications for any changes made to the warehoused SPSS data.

#### Phase III

We will flag inconsistencies for further discussion and, if decisions are made to adjust reported values, those decision rules will be fully documented for reporting purposes. These rules will be informed by the literature, What Works Clearinghouse (WWC) standards, or best practice guidance. We will check for within-time point and across-time point inconsistencies and set up cleaning rules for both. For example, within a time point (eg, baseline), if a participant reports no history of anal sex but reports using a condom during anal sex in the last 3 months, both responses would be edited as missing values. Similarly, across time points (ie, baseline, 3-month, and 9-month assessments), if someone reports that they have never had anal sex at 9 months, but report having had anal sex at the 3-month evaluation, we will carry out two sensitivity analyses with (1) recoded 3-month data to match 9-month data and (2) recoded 9-month data to match 3-month data [37]. However, it is important to note that such inconsistencies are not expected due to the built-in skip patterns in our Qualtrics survey but are planned for nonetheless.

Additionally, any response values that were not supposed to have been provided based on built-in skip patterns will be recoded as “not applicable.” Overall, missing values will be differentiated with appropriate coding as “nonresponse,” “do not know,” and “not applicable” as needed, and some anomalies (if plausible) will be left unchanged (eg, true extreme values such as age at 18 years). Finally, original respondents’ data will be kept as a backup, and we will explore sensitivity analyses to check to what extent data cleaning edits influence our results, including the use of multiple imputation procedures described below.

### Primary Outcome

The primary outcome of this study is the impact of About Us relative to the standard of care 9 months following the end of the intervention on the prevalence of vaginal or anal sex without condoms in the past 3 months. Items specifically examine condom use during vaginal and anal sex independently (Textbox 1).

### Secondary Outcomes

The secondary outcomes are four-fold: 3 months and 9 months following the end of the intervention, what is the impact of About Us relative to the standard of care on (1) the prevalence of abstinence from vaginal or anal sex in the past 3 months, (2) composite scores of relationship communication and positive conflict resolution among participants involved in a relationship at baseline, (3) the prevalence of school-based health center service use or information receipt in the past 3 months, and (4) composite scores of condom use intentions and attitudes regarding condoms and other types of birth control? These secondary outcomes are summarized in Tables S1-S4 in Multimedia Appendix 1.

### Generalities of Statistical Analysis Methods

Statistical analysis will be undertaken using SPSS and SAS. All participants randomized to either intervention (About Us) or BAU will be included in the analyses using the intent-to-treat (ITT) principles. Our benchmark analysis data set will include all randomized participants with imputed data for missing covariate and outcome variables.

As part of our sensitivity analyses, two additional types of analyses will be implemented: modified intent-to-treat (modified ITT) and complete case analysis.

Modified ITT analysis (ie, analysis based on a data set that includes all randomized subjects who provide baseline measurements on primary and secondary outcomes and have at least one follow-up assessment) will also be implemented under different conditions for missing data adjustment (described below).

### Assessment of Baseline Equivalence

Equivalence between the intervention and control groups will be assessed for demographic characteristics and primary and secondary outcomes at 3 time points: baseline, 3-month, and 9-month follow-up. At the 3-month and 9-month time points, baseline characteristics and outcomes will be compared between participants who completed each assessment separately. For example, if 400 out of 533 randomized participants completed the 3-month assessment, their baseline characteristics and outcome measures will be compared between intervention and control group participants (n=400). The baseline equivalence results will be used to help identify issues such as a potential lack of equivalence due to attrition (study or program attrition). Baseline demographic characteristics and primary and secondary outcome measures that are statistically different between the treatment and control groups will be controlled in our primary and secondary outcome analyses as described below.

Demographic characteristics of interest include age, sex assigned at birth, and race and ethnicity. The mean age and its corresponding SD for intervention and control groups will be calculated. Frequencies and proportions will be produced for categorical outcomes such as sex assigned at birth and race and ethnicity. Race will be categorized into non-Hispanic White, non-Hispanic Black, Hispanic, and other races.

A Pearson’s Chi-square test (or Fisher’s exact test as needed) will be used to examine baseline differences between categorical variables (sex assigned at birth, race and ethnicity, primary and secondary outcomes, and group assignment at baseline, 3-month, and 9-month assessment time points separately.

A two-sample independent t-test (or a Mann-Whitney test, nonparametric test as needed) will be used to examine baseline differences between continuous variables (age and secondary outcomes) and group assignment at baseline, 3-month, and 9-month assessment time points separately.

Baseline equivalence analyses will be conducted using SAS, and statistical significance will be assessed at an alpha (Type I error) of 5%.

### Preliminary Data Analysis

Preliminary data analysis will involve routine range checks for continuous variables and frequencies and cross-tabulations for categorical variables. If necessary, continuous outcomes (eg, score-based measures) will be corrected using the least powerful transformations possible to meet our statistical modeling assumptions (ie, “normalize” univariate data that might be skewed or “straighten out” a bivariate curvilinear relationship) of outcome and covariate relationships for linear regression [38]. In addition, bivariate analyses will be performed to identify potential nonlinear relationships (eg, between 9-month continuous outcomes and baseline characteristics and measures such as age and composite scores) that may need to be modeled. Preliminary analysis of score-based (or instrument-based) outcomes will include examining evidence of construct validity and reliability. Construct validity refers to the degree to which an instrument or measure assesses the underlying theoretical construct it is supposed to measure (ie, the test is measuring what it is purported to measure). This will be examined via confirmatory factor analysis for score-based outcomes. Reliability refers to the degree of interrelationship or homogeneity among question items on a test (questionnaire), such that they are consistent with one another and measure the same construct; this will be examined by generating an internal consistency index–Cronbach’s alpha for our score-based outcomes.

### Statistical Analysis Models

For dichotomous outcomes at 9 months, we will use logistic regression models with covariates (see Textbox 2) for the intervention, baseline outcome variables, strata variables (sex assigned at birth and number of screening experience factors present), sociodemographic characteristics (age and race and ethnicity), the time elapsed between baseline and the follow-up survey (at 3-month and 9-month follow-up), cohort, and school. For continuous outcomes, we will use linear regression models with covariates for the intervention, baseline outcome variables, strata variables (sex and number of screening risk factors present), sociodemographic characteristics (age and race and ethnicity), the time elapsed between baseline and follow-up surveys (at 3-month and 9-month follow-up), cohort, and school. Additional (exploratory) analyses will test for 3-way and 2-way statistical interactions and adjust for prognostic factors such as potential confounders of intervention effects. Similarly, we will include a covariate that captures whether our outcome data were collected pre-COVID-19 versus during the COVID-19 pandemic. Potential 2-way interactions involving this covariate and intervention group, age and grade, and relationship status will be examined to test the differential effects of the pandemic on outcomes of interest.

**Covariates included in impact analyses.**

**Screening factor:** one or two screening factors present (categorical variable).

**Age (years):** baseline date to DOB (continuous variable).

**Sex:** sex assigned at birth; 1=male, 2=female (dichotomous variable).

**Race and ethnicity:** White, Black, Hispanic, other (categorical variable).

**Number of days between baseline and the follow-up survey (3 or 9 months):** follow-up date to baseline date (continuous variable).

**Cohort:** cohort 1 (spring 2018), cohort 2 (fall 2018), cohort 3 (spring 2019), cohort 4 (fall 2019), and cohort 5 (spring 2020; categorical variable).

**School:** high schools 1 to 7.

We will also take advantage of all measurement time point data and appropriately model the data hierarchy by exploring the use of generalized linear mixed models (GLMM) [39,40] to evaluate intervention effectiveness. The GLMM will allow us to use all data available and adjust for multilevel dependencies (eg, repeated measures, such as baseline and follow-up at 3 months and 9 months, within an individual participant nested within the intervention or control group, within a cohort (block) and study site). Additional analyses to account for multilevel effects will be explored as secondary or sensitivity analyses.

#### Presentation of Continuous Outcomes

Least squares (LS) means, corresponding SEs, 95% 2-sided CIs, and 2-tailed *P* values will be presented for the within-group (ie, intervention and control) outcome measures. For each between-group, the difference in LS means, corresponding SE, 2-sided 95% CI, and 2-tailed *P* value will also be derived from the linear regression model and presented. Standard model diagnostics will be performed to assess the validity of the proposed model. These diagnostics will include examining the residuals for normality and homoscedasticity as well as testing for the significance of the intervention by baseline outcome interaction terms.

#### Presentation of Categorical Outcomes

The estimated odds ratio, SE, 95% Wald CI, and *P* value will be presented for each between-group comparison of interest. Standard model diagnostics will be performed to assess the validity of the proposed model. These diagnostics will include testing the goodness of fit with the Hosmer-Lemeshow test and examining influence statistics for potentially outlying observations. In addition, the number and percentage of subjects with a primary outcome (eg, condomless vaginal or anal sex) will be presented for each treatment group, including model estimated probabilities and statistical significance of differences observed (at α=.05).

### Handling Missing Data

Our general approach to missing data will involve taking advantage of all observed information while not exaggerating the precision of findings based on incomplete data [41,42].

All variables described for our final regression models will be used in our imputation procedures. That is, outcome measures (at 3-month and 9-month follow-up analyzed separately), treatment condition, baseline outcome variables, strata variables (sex and number of screening experience factors present), sociodemographic characteristics (age, race, and ethnicity), the time elapsed between baseline and follow-up survey (at 3-month or 9-month follow-up), and school. The treatment and control group participant data will be imputed separately [43].

Multivariate imputation by chained equations [44] methods using PROC MI and MIANALYZE procedures in SAS will be used to create multiple imputations (replacement values) for multivariate missing data (eg, continuous, binary, unordered categorical, and ordered categorical data) based on a fully conditional specification [45], where each incomplete variable is imputed by a separate model [46]. All missingness (nonresponse, program attrition, or study loss to follow-up) will be treated the same way for our benchmark analysis. Additional analyses (see Sensitivity Analysis) will be explored where reasons for program attrition are accounted for in the generation of imputed covariate and outcome values. All variables (ie, outcome and baseline characteristics) described in the above analysis models will be used for the imputation procedures. The number of data sets to be imputed will be determined using the quadratic rule recommended by von Hippel [47]. In addition to the baseline value of the outcome of interest, age, race and ethnicity, the sex assigned at birth, and the number of screening experience factors (1 or 2; ie, adolescents had either exposure to violence or a prior or current experience with a romantic relationship or both), and time (days) between baseline and 3-month or 9-month follow-up will be included in our final analysis models (ie, linear and logistic regression).

### Sensitivity Analysis

In addition to the benchmark analyses described above (3-month and 9-month follow-up examined separately), two types of analyses (ie, complete case analysis—no imputation for missing data—and modified ITT with imputed data) will be implemented using procedures similar to the benchmark analyses (linear and logistic regression models). The robustness of the inferential findings will be assessed by comparing differences in analytical findings across the three types of analyses (ITT with imputed data, complete case analysis, and modified ITT).

## Results

Baseline data collection commenced in February 2018 and was completed in March 2020, yielding 5 cohorts and 533 study participants, with 316 assigned to the intervention group and 217 assigned to the control group. Though the project team anticipated an additional semester of implementation in the fall of 2020, the COVID-19 pandemic and subsequent school closures interfered with the scheduling of additional programming. We continued with online data collection and anticipate the completion of follow-up data collection by May 2021.

## Discussion

### Support for the Intervention

Most of the existing EBPs yield relatively short-term gains [48] and were developed using normative decision-making models [49]. These models describe decision-making as a deliberate and analytic process and are useful for predicting behaviors that are typically unemotional [50]; the utility of these models is limited for sexual behaviors, which are inherently emotional. Findings stemming from developmental neuroscience experts suggest that changes in relational, emotional, and social processing play a critical role in influencing adolescent behavior, highlighting the potential of integrating emotionally relevant learning strategies into sexual health programs; by doing so, the content becomes more meaningful and relevant to adolescents, and better supports the development of decision-making skills [51]. Further, most existing interventions are inherently cognitive, teaching adolescents how to refuse unwanted or unprotected sexual intercourse, but do not address the circumstances or situations under which adolescents might be willing to engage in certain sexual behaviors [51]. About Us draws on this body of research to create a more contextually and developmentally relevant program that addresses motivation and emotional influences in sexual decision-making.

The lack of student interactivity is a major pedagogical issue facing learning environments today [52]. EBPs for teen pregnancy and STI prevention share similar interactive instructional strategies (eg, mini-lectures, games, role-playing, and simulations); however, other strategies could extend the program’s impact. Strategies include storytelling and the use of blended learning. Stories have been recognized for centuries as a powerful tool for organizing and transmitting information [53]. They are one way to pique students’ curiosity and build interest while framing new concepts, illustrating consequences, modeling skills, and providing context. Neuroscience supports the use of stories as anchors of information assisting in the learning process [54]. In addition, educators now emphasize the importance of blended learning, which incorporates the use of new online technologies in face-to-face settings to engage students in active and interactive learning [55]. The potential of using technology in changing sexual behaviors is highlighted in research with computer-based or blended learning programs [56,57].

Further, technology provides young people with opportunities to gain virtual experiences related to relationships. Some research suggests there is an association between emotional experience gained in virtual environments with emotional experiences lived in real-life contexts, which can provide a safe and low-risk venue for emotional learning [58]. We draw on both of these strategies in this project.

### Limitations

This study is not without limitations. The COVID-19 global pandemic severely impacted this project. As a result, policies enacted by state and local governments, by school sites participating in About Us, and by the research institutions conducting the evaluation were implemented to protect as many people as possible from the virus. This included suspending in-person intervention delivery, recruitment, and data collection and necessitated the transition to online-only follow-up survey administrations beginning in March 2020. These changes reduced the number of students recruited and prevented those who had already been recruited from receiving the intervention.

Implementation of the intervention was also impacted by instances of turnover within schools and health centers, which sometimes confused all stakeholders. For example, new administrators frequently had no knowledge of the research study because it had not been communicated to them by the outgoing administrators. Similarly, new health center staff were often overwhelmed by “inheriting” this new program from a predecessor, thus slowing the pace of required implementation and evaluation activities. These instances of turnover combined with the challenges of scheduling intervention delivery to occur during the already limited regular school day or after school resulted in lower enrollment in the study.

Another limitation was the incompleteness of the contact information provided by adolescents participating in the study. For example, in some instances, adolescents either initially provided incorrect information or did not provide updated information for study team members to use for follow-up contact attempts. This reduced our follow-up survey response rate and resulted in participants being lost to follow-up. When possible, study team members worked with SBHC staff to reach students with outdated or incorrect contact information and encourage them to participate in scheduled survey administration.

The study was also impacted by a lower-than-expected number of returned consent forms, which resulted in lower-than-expected enrollment. SBHC staff utilized various methods for distributing the consent forms (eg, individually to each student during screening visits or in a welcome packet for parents at the beginning of school term), but the collection of consent forms may have been negatively impacted by lack of follow-up with adolescents about returning the forms to the SBHC. While our study team employed methods previously shown to be associated with improved consent form return in youth samples (eg, providing incentives for consent form return or utilizing school staff for consent form collection) [59,60], additional approaches such as greater engagement with parents or using an opt-out versus an opt-in approach where appropriate may improve consent form return rates.

In some cases, adolescents transferred to different school sites that were not involved in the study. In these instances, we could not rely on SBHC staff to reach out to adolescents if their contact information was incorrect or incomplete, and they were lost to follow-up.

## Appendix

Multimedia Appendix 1 Supplementary tables.

## Abbreviations

BAU: business as usual
CSHA: California School-Based Health Alliance
EBP: evidence-based practice
EDC: evaluation data collector
GLMM: generalized linear mixed model
ITT: intent-to-treat
LS: least squares
OR: odds ratio
PREIS: Personal Responsibility Education Innovative Strategies
PYD: positive youth development
RCT: randomized controlled trial
SBHC: school-based health center
STI: sexually transmitted infections
