# Supplementary material for: Promoting Adolescent Healthy Relationships (The About Us Program): Protocol for a Randomized Clinical Trial
Source: JMIR Res Protoc. 2021 Sep 1;10(9):e30499. doi: 10.2196/30499 (PMC8444045; doi:10.2196/30499)
Supplement: Multimedia Appendix 1 [file resprot_v10i9e30499_app1.docx]

Multimedia Appendix 1

**Table S1.** Outcomes used for secondary research questions (2.1. Prevalence of abstinence from vaginal or anal sex in the past 3 months?)

| **Outcome name** | **Source item(s)** | **Constructed measure** | **Timing of measure** |
| --- | --- | --- | --- |
| Abstinence from vaginal or anal sex in the past 3 months  (Core Measures for PREIS Grantees ) [36] | In the past 3 months, how many times did you have vaginal sex? *Vaginal sex is when a penis is put in a vagina.*  In the past 3 months, how many times did you have anal sex? *Anal sex is when a penis is put in a rectum, ass, or butt.*  Original response options for vaginal or anal sex include:  1= I have never had vaginal sex 2 = I have had vaginal sex, but not in the past 3 months 3 = Once  4= 2-3 times 5 = 4 or more times  1= I have never had anal sex 2 = I have had anal sex, but not in the past 3 months 3 = Once  4= 2-3 times 5 = 4 or more times | Construct a single, dichotomous outcome coded as 0 if the respondent indicated they had vaginal or anal sex in the past 3 months, 1 if they did not, and missing otherwise.  To be recoded into a single dichotomous variable (vaginal or anal sex):  0 = Had vaginal or anal sex in past 3 months (3, 4 or 5).    1 = No vaginal or anal sex in the past 3 months (1 or 2)  . = Missing response for vaginal or anal sex in past 3 months | 3 months and 9 months following completion of the program. |

**Table S2.** Outcomes used for secondary research questions (2.2. Composite scores of relationship communication and positive conflict resolution?)

| **Outcome name** | **Source item(s)** | **Constructed measure** | **Timing of measure** |
| --- | --- | --- | --- |
| Relationship Communication  (Taylor & Mumford, 2016) [30] | Thinking about your most recent partner (which may be a current partner), how much do you agree or disagree with the following statements? | A composite score will be constructed by adding up the values associated with each question item. For the composite score, a lower total score is desired and captures positive relationship and conflict resolution communication relative to a higher total score. However, in analyses, we will recode the composite score, such that a higher total score is desired and captures positive relationship and conflict resolution communication relative to a lower total score.  Total scores are expected to range between 0 and 64 derived from summing the values from 14 question items.  We will calculate score reliability and assess evidence of validity (i.e., factor analysis), at baseline, for the data collected via the scale.  Missing values will be imputed for question items using methods described below prior to the generation of total scores.  Restrict the analytic sample to only those participants who had reported being in a relationship (at baseline).participants | 3 and 9 months following completion of the program. |
|  | Your partner sometimes wants to control what you do.  A. Strongly disagree (value=0) B. Disagree (value=1) C. Neutral (value=2) D. Agree (value=3) E. Strongly agree (value=4) |  | 3 and 9 months following completion of the program. |
|  | Your partner always tries to change you.  A. Strongly disagree (value=0) B. Disagree (value=1) C. Neutral (value=2) D. Agree (value=3) E. Strongly agree (value=4) |  | 3 and 9 months following completion of the program. |
|  | Your partner expects you to respond immediately to texts or phone calls.  A. Strongly disagree (value=0) B. Disagree (value=1) C. Neutral (value=2) D. Agree (value=3) E. Strongly agree (value=4) |  | 3 and 9 months following completion of the program. |
|  | You sometimes try to control what your partner does.  A. Strongly disagree (value=0) B. Disagree (value=1) C. Neutral (value=2) D. Agree (value=3) E. Strongly agree (value=4) |  | 3 and 9 months following completion of the program. |
|  | You always try to change your partner.  A. Strongly disagree (value=0) B. Disagree (value=1) C. Neutral (value=2) D. Agree (value=3) E. Strongly agree (value=4) |  | 3 and 9 months following completion of the program. |
|  | Sometimes you don't know quite what to say with your partner.  A. Strongly disagree (value=0) B. Disagree (value=1) C. Neutral (value=2) D. Agree (value=3) E. Strongly agree (value=4) |  | 3 and 9 months following completion of the program. |
|  | You would be uncomfortable having intimate conversations with your partner.  A. Strongly disagree (value=0) B. Disagree (value=1) C. Neutral (value=2) D. Agree (value=3) E. Strongly agree (value=4) |  | 3 and 9 months following completion of the program. |
|  | Sometimes you find it hard to talk about your feelings with your partner.  A. Strongly disagree (value=0) B. Disagree (value=1) C. Neutral (value=2) D. Agree (value=3) E. Strongly agree (value=4) |  | 3 and 9 months following completion of the program. |
|  | Sometimes you feel you need to watch what you say to your partner.  A. Strongly disagree (value=0) B. Disagree (value=1) C. Neutral (value=2) D. Agree (value=3) E. Strongly agree (value=4) |  | 3 and 9 months following completion of the program. |
|  | Your partner cares about you.  A. Strongly disagree (value=4) B. Disagree (value=3) C. Neutral (value=2) D. Agree (value=1) E. Strongly agree (value=0) |  | 3 and 9 months following completion of the program. |
|  | Your partner and you are practically inseparable.  A. Strongly disagree (value=4) B. Disagree (value=3) C. Neutral (value=2) D. Agree (value=1) E. Strongly agree (value=0) |  | 3 and 9 months following completion of the program. |
|  | You would rather be with your partner than anyone else.  A. Strongly disagree (value=4) B. Disagree (value=3) C. Neutral (value=2) D. Agree (value=1) E. Strongly agree (value=0) |  | 3 and 9 months following completion of the program. |
|  | You are very attracted to your partner.  A. Strongly disagree (value=4) B. Disagree (value=3) C. Neutral (value=2) D. Agree (value=1) E. Strongly agree (value=0) |  | 3 and 9 months following completion of the program. |
|  | Your partner always seems to be on your mind.  A. Strongly disagree (value=4) B. Disagree (value=3) C. Neutral (value=2) D. Agree (value=1) E. Strongly agree (value=0) |  | 3 and 9 months following completion of the program. |
| Positive Conflict Resolution  (Adapted from Arellano & Markman, 1995) [31] | When my partner and I disagree or have a misunderstanding I talked about it with them.  A. Never or almost never (value=4) B. Some of the time (value=3) C. Most of the time (value=2) D. Always or almost always (value=1) E. This hasn't happened to me (value=0) | A composite score will be constructed by adding up the values associated with each question item. For the composite score, a lower total score is desired and captures positive conflict resolution relative to a higher total score. However, in analyses, we will recode the composite score, such that a higher total score is desired and captures positive relationship and conflict resolution communication relative to a lower total score.  Total scores are expected to range between 5 and 20 derived from summing the values from 5 question items. We will code items in which respondents marked “This hasn’t happened to me” as missing.  We will calculate score reliability and assess evidence of validity (i.e., factor analysis), at baseline, for the data collected via the scale.  Missing values will be imputed for question items using methods described below prior to the generation of total scores.  Restrict the analytic sample to only those participants who had reported being in a relationship (at baseline).  5-item-scale score participants | 3 and 9 months following completion of the program. |
|  | I tell my partner when they have done something that bothers me.  A. Never or almost never (value=4) B. Some of the time (value=3) C. Most of the time (value=2) D. Always or almost always (value=1) E. This hasn't happened to me (value=0) |  | 3 and 9 months following completion of the program. |
|  | I show interest in my partner's activities.  A. Never or almost never (value=4) B. Some of the time (value=3) C. Most of the time (value=2) D. Always or almost always (value=1) E. This hasn't happened to me (value=0) |  | 3 and 9 months following completion of the program. |
|  | When an issue arises, I ask my partner directly how they feel or think about it.  A. Never or almost never (value=4) B. Some of the time (value=3) C. Most of the time (value=2) D. Always or almost always (value=1) E. This hasn't happened to me (value=0) |  | 3 and 9 months following completion of the program. |
|  | When an issue arises, my partner and I discuss it and then we move on.  A. Never or almost never (value=4) B. Some of the time (value=3) C. Most of the time (value=2) D. Always or almost always (value=1) E. This hasn't happened to me (value=0) |  | 3 and 9 months following completion of the program. |

**Table S23** Outcomes used for secondary research questions (2.3. Prevalence of school-based health center service use or information receipt in the past 3 months?)

| **Outcome name** | **Source item(s)** | **Constructed measure** | **Timing of measure** |
| --- | --- | --- | --- |
| SBHC Utilization  (Cleland, 2001) [32] | Have you ever visited your school-based health center (SBHC) to receive services or information on contraception, pregnancy, abortion or sexually transmitted diseases?  A. No B. Yes | Construct a single, dichotomous outcome coded as:  1 if Yes[B] - you ever visited your school-based health center (SBHC) to receive services or information on contraception, pregnancy, abortion or sexually transmitted diseases and the number of times  services/information was sought in the past 3 months is greater than 0,  0 if they never visited SBHC OR did not seek services/information from SBHC in the last 3 months, and missing otherwise. | 3 and 9 months following completion of the program. |
|  | How many times have you sought services or information from the SBHC for these services in the last 3 months?   1. ___ number of times 2. Did not seek care in last 3 months |  |  |

**Table S4.** Outcomes used for secondary research questions (2.4. Composite scores of condom intentions and attitudes regarding condoms and other birth control?)

| **Outcome name** | **Source item(s)** | **Constructed measure** | **Timing of measure** |
| --- | --- | --- | --- |
| Behavioral Intentions - Condoms  (Escribano et al., 2016) [33] | I will get condoms if I need them.  A. Definitely not B. Probably not C. Possibly D. Probably E. Definitely | A composite score will be constructed by adding up the values associated with each question item. For the composite score, a higher total score is desired and captures a stronger condom intention relative to a lower total score.  Total scores are expected to range between 0 and 12 derived from summing the values from 3 question items.  We will calculate score reliability and assess evidence of validity (i.e., factor analysis), at baseline, for the data collected via the scale.  Missing values will be imputed for question items using methods described below prior to the generation of total scores.  The analytic sample will be all respondents.  3-item scale score | 3 and 9 months following completion of the program. |
|  | I will talk about condom use with my sexual partner before having sex.  A. Definitely not B. Probably not C. Possibly D. Probably E. Definitely |  | 3 and 9 months following completion of the program. |
|  | I will use condoms when I have sex.  A. Definitely not B. Probably not C. Possibly D. Probably E. Definitely |  | 3 and 9 months following completion of the program. |
| Multidimensional Condom Attitudes Scale (MCAS)  (Starosta et al., 2014) [34] | Condoms are an effective method of preventing the spread of AIDS and other sexually transmitted diseases.  A. Strongly disagree (value=0) B. Disagree (value=1) C. Neutral (value=2) D. Agree (value=3) E. Strongly agree (value=4) | A composite score will be constructed by adding up the values associated with each question item. For the composite score, a higher total score is desired and captures more positive attitude towards condoms, relative to a lower total score.  Total scores are expected to range between 0 and 12 derived from summing the values from 3 question items.  We will calculate score reliability and assess evidence of validity (i.e., factor analysis), at baseline, for the data collected via the scale.  Missing values will be imputed for question items using methods described below prior to the generation of total scores.  The analytic sample will be all respondents. | 3 and 9 months following completion of the program. |
|  | Condoms are an excellent means of birth control/contraception.  A. Strongly disagree (value=0) B. Disagree (value=1) C. Neutral (value=2) D. Agree (value=3) E. Strongly agree (Value=4) |  | 3 and 9 months following completion of the program. |
|  | Condoms do not offer reliable protection against sexually transmitted diseases.  A. Strongly disagree (value=0) B. Disagree (value=1) C. Neutral (value=2) D. Agree (value=3) E. Strongly agree (value=4)  (*Will be reverse coded*) |  | 3 and 9 months following completion of the program. |
| Contraceptive Attitude Scale  (Kyes, 1998) [35] | Birth control/contraceptives are not really necessary unless a couple has engaged in intercourse more than once.  A. Strongly disagree (value=0) B. Disagree (value=1) C. Neutral (value=2) D. Agree (value=3) E. Strongly agree (value=4)  (*Will be reverse coded*) | A composite score will be constructed by adding up the values associated with each question item. For the composite score, a higher total score is desired and captures positive attitudes towards contraceptives, relative to a lower total score.  Total scores are expected to range between 0 and 44 derived from summing the values from 11 question items.  We will calculate score reliability and assess evidence of validity (i.e., factor analysis), at baseline, for the data collected via the scale.  Missing values will be imputed for question items using methods described below prior to the generation of total scores.  The analytic sample will be all respondents. | 3 and 9 months following completion of the program. |
|  | I would not have vaginal intercourse if no birth control/contraceptive method was available.  A. Strongly disagree (value=0) B. Disagree (value=1) C. Neutral (value=2) D. Agree (value=3) E. Strongly agree (value=4) |  | 3 and 9 months following completion of the program. |
|  | Using birth control/contraceptives is a way of showing that you care about your partner.  A. Strongly disagree (value=0) B. Disagree (value=1) C. Neutral (value=2) D. Agree (value=3) E. Strongly agree (value=4) |  | 3 and 9 months following completion of the program. |
|  | People should use birth control/contraceptives regardless of how long they have known their sexual partner.  A. Strongly disagree (value=0) B. Disagree (value=1) C. Neutral (value=2) D. Agree (value=3) E. Strongly agree (value=4) |  | 3 and 9 months following completion of the program. |
|  | Birth control/contraceptives are difficult to obtain.  A. Strongly disagree (value=0) B. Disagree (value=1) C. Neutral (value=2) D. Agree (value=3) E. Strongly agree (value=4)  (*Will be reverse coded*) |  | 3 and 9 months following completion of the program. |
|  | Birth control/contraceptives can actually make intercourse seem more pleasurable.  A. Strongly disagree (value=0) B. Disagree (value=1) C. Neutral (value=2) D. Agree (value=3) E. Strongly agree (value=4) |  | 3 and 9 months following completion of the program. |
|  | I would feel more relaxed during sex if birth control/contraceptive method is used.  A. Strongly disagree (value=0) B. Disagree (value=1) C. Neutral (value=2) D. Agree (value=3) E. Strongly agree (value=4) |  | 3 and 9 months following completion of the program. |
|  | In the future, I plan to use contraceptives any time I have vaginal sex.  A. Strongly disagree (value=0) B. Disagree (value=1) C. Neutral (value=2) D. Agree (value=3) E. Strongly agree (value=4) |  | 3 and 9 months following completion of the program. |
|  | I would use birth control/contraception even if my partner did not want me to.  A. Strongly disagree (value=0) B. Disagree (value=1) C. Neutral (value=2) D. Agree (value=3) E. Strongly agree (value=4) |  | 3 and 9 months following completion of the program. |
|  | Using birth control/contraceptives makes a relationship seen too permanent.  A. Strongly disagree (value=0) B. Disagree (value=1) C. Neutral (value=2) D. Agree (value=3) E. Strongly agree (value=4)  (*Will be reverse coded*) |  | 3 and 9 months following completion of the program. |
|  | Birth control/contraceptives make intercourse seem too planned.  Strongly disagree (value=0)B. Disagree (value=1) C. Neutral (value=2) D. Agree (value=3) E. Strongly agree (value=4)  (*Will be reverse coded*) |  | 3 and 9 months following completion of the program. |
